# Roles and potential applications of non-coding RNAs in cancer treatment with immune checkpoint inhibitors and immunomodulatory therapies

**DOI:** 10.20517/cdr.2025.213

**Published:** 2026-04-15

**Authors:** Yayu Chen, Zhishuang Ye, Yanping Wang, Shanlan Liang, Daniel Xin Zhang

**Affiliations:** ^1^Department of Health Sciences, Hong Kong Metropolitan University, Hong Kong, China.; ^2^School of Nursing and Health Sciences, Hong Kong Metropolitan University, Hong Kong, China.; ^3^Hong Kong Metropolitan University Shenzhen Research Institute, Shenzhen 518057, Guangdong, China.

**Keywords:** Non-coding RNA, cancer, therapy, immunotherapy, immune checkpoint inhibitors

## Abstract

Non-coding RNAs (ncRNAs) have emerged as key regulators of cancer–immune crosstalk, especially in an era when immune checkpoint inhibitors and other immunomodulatory therapies are reshaping the cancer treatment landscape. Accumulating evidence continues to indicate that ncRNAs, including microRNAs, long non-coding RNAs and circular RNAs, critically connect oncological signaling with immune interactions, thereby influencing clinical outcomes. In this review, we summarize how ncRNAs modulate key immune checkpoint axes, particularly programmed cell death protein 1 (PD-1)/programmed cell death ligand 1 (PD-L1) and cytotoxic T-lymphocyte antigen 4 (CTLA-4). We also discuss ncRNA networks that are actively involved in modern cancer immunotherapies, such as T cell–based therapies, macrophage and dendritic cell engineering, cytokine therapies, cancer vaccines and oncolytic viruses. Building on these mechanistic insights, we outline the potential of ncRNAs as biomarkers for predicting response and prognosis, as future therapeutic targets to improve and enhance immunotherapy combinations, along with key barriers in the field and emerging solutions. Altogether, the evidence not only highlights ncRNAs as rising stars in precision immuno-oncology, but also motivates future opportunities to incorporate ncRNAs into clinical consideration.

## INTRODUCTION

### The multidimensional evolution of cancer immunotherapy: current status and challenges

In recent years, cancer immunotherapy has fundamentally reshaped the therapeutic paradigm of oncology through multidimensional strategies, including immune checkpoint inhibitors (ICIs), cell therapies, and more, establishing itself as a revolutionary breakthrough following surgery, radiotherapy, chemotherapy, and targeted therapy. By alleviating immunosuppression, ICIs have markedly improved clinical outcomes in advanced-stage malignancies, a finding corroborated by substantial clinical evidence^[1,2]^. The 10-year follow-up findings of the KEYNOTE-006 trial (NCT01866319) confirm that pembrolizumab delivers a significant, sustained survival benefit compared with ipilimumab in patients with advanced melanoma, with 10-year overall survival rates of 34.0% and 23.6% respectively. These findings offer long-term prospective follow-up data to support programmed cell death protein 1 (PD-1) inhibitor use in this clinical setting^[3]^. This unequivocal survival benefit in select advanced cancers has solidified the standard-of-care status of this class of agents. Chimeric antigen receptor (CAR)-T cell therapy has exhibited impressive therapeutic efficacy in hematological malignancies^[4]^; for instance, in relapsed/refractory diffuse large B-cell lymphoma (DLBCL), CAR-T therapy has been established as a standard treatment option for patients with early relapsed or refractory disease^[5]^. T-cell receptor (TCR)-T therapy, through genetic modification to target antigens such as New York esophageal squamous cell carcinoma 1 (NY-ESO-1), has broadened the therapeutic scope^[6]^. Tumor vaccines have undergone successive technological iterations; personalized neoantigen vaccines, informed by whole-exome sequencing to identify tumor mutational burden, have shown efficacy in early-stage melanoma trials^[7]^. Oncolytic viruses (OVs), leveraging a dual mechanism of action - direct tumor lysis coupled with activation of the Stimulator of Interferon Genes (STING) pathway - have demonstrated a favorable safety profile in high-grade gliomas^[8]^.

Nevertheless, the field confronts formidable challenges. ICIs are associated with primary or acquired resistance, and cardiotoxicity has been reported in patients receiving ICI treatment, with a 40% rate of major adverse cardiovascular events in patients diagnosed with ICI-related myocarditis^[9,10]^. These agents can also precipitate multi-organ inflammation and, in some cases, fatal complications^[11-14]^. Investigators have observed that in patients with advanced melanoma undergoing ICI therapy, immune-related adverse events (irAEs)-particularly endocrine and gastrointestinal toxicities can impact overall survival^[15]^. CAR-T therapy faces hurdles in solid tumors due to the tumor microenvironment (TME), exemplified by resistance induced by the transforming growth factor-β (TGF-β) pathway and the suppression of T-cell function by myeloid-derived suppressor cells, compounded by T-cell exhaustion^[16]^. Tumor vaccines are constrained by low antigen immunogenicity, short half-lives, susceptibility to degradation, and limitations imposed by human leukocyte antigen (HLA) restriction^[17]^. OVs encounter technical barriers, including nonspecific sequestration by the lungs, liver, and spleen following intravenous administration, which results in suboptimal tumor-targeting doses^[18]^.

Despite the transformative clinical success of modern immunotherapy, the persistence of resistance, toxicity, and limited efficacy in many tumor types underscores the need to better understand the molecular determinants of antitumor immune responses. In this context, attention has increasingly shifted toward regulatory layers beyond conventional protein-coding genes. Non-coding RNAs (ncRNAs), as versatile post-transcriptional and epigenetic regulators, have emerged as key players in shaping immune cell function, tumor immune evasion, and responses to immunotherapy. This evolving insight provides a logical foundation for examining their pivotal role in cancer immune regulation.

### The pivotal role of ncRNAs in cancer immune regulation

ncRNAs have emerged as key regulators of cancer immunity and as promising mechanistic links between tumor biology and immunotherapeutic response. They can be classified into two major categories based on their biological functions: housekeeping ncRNAs and regulatory ncRNAs. Housekeeping ncRNAs, which are fundamental components of cellular life processes, are generally evolutionarily conserved. These include ribosomal RNA (rRNA), which participates in the core process of genetic information transfer, and transfer RNA (tRNA), which is involved in protein synthesis. Additionally, small nuclear RNA (snRNA, 60-300 nt) and small nucleolar RNA (snoRNA, 60-300 nt), which play roles in RNA processing, ensure the normal functioning of cells by maintaining ribosome assembly, protein translation, and RNA splicing. In contrast, regulatory ncRNAs, such as microRNAs (miRNAs), long non-coding RNAs (lncRNAs), and circular RNAs (circRNAs), have been reported to dynamically regulate the TME. miRNAs regulate key immune checkpoint molecules through base-pairing interactions, modulating processes such as T cell activation, differentiation, and B cell antibody production^[19]^. lncRNAs can act as endogenous competing RNAs (ceRNAs), inhibiting miRNA function and thus regulating immune responses. By binding to miRNAs, lncRNAs reduce the interaction between miRNAs and their target messenger RNAs (mRNAs), further suppressing miRNA activity^[20]^. circRNAs, owing to their structural stability, participate in alterations of the tumor-infiltrating lymphocyte (TIL) immune microenvironment in cancer^[21]^. For example, lncRNA HOX antisense intergenic RNA (HOTAIR) promotes immune evasion^[22]^, while circRNA-002178 elevates the expression of programmed cell death ligand 1 (PD-L1)/PD-1 in cancer cells and T cells, directly intervening in core mechanisms of immunotherapy^[23]^. Given the targeting abilities and significant laboratory and clinical evidence, this review will focus on these regulatory ncRNAs.

Immune cell function serves as the central driver of anti-tumor responses, while the immune-suppressive status of the TME and resistance mechanisms represent key barriers to therapeutic success. At the immune cell level, ncRNAs precisely regulate differentiation and functional polarization. Deficiency of miR-7 results in the upregulation of mitogen-activated protein kinase 4 (MAPK4), which activates downstream signaling pathways [AKR mouse T-cell lymphoma virus oncogene (Akt)/extracellular signal-regulated kinase (ERK)/nuclear factor kappa B (NF-κB)], promoting T helper 1 cell (Th1 cell) polarization^[24]^. In glioma cells, the circRNA circular MAPK4 (circMAPK4) has been shown to inhibit apoptosis and drive tumor progression via sequestering miR-125a-3p, which in turn regulates the p38 mitogen-activated protein kinase (p38)/mitogen-activated protein kinase (MAPK) signaling cascade^[25]^. circ_0000190 in multiple myeloma indirectly regulates MAPK4 by sequestering miR-767-5p, which in turn inhibits tumor cell proliferation and induces apoptosis^[26]^. In breast cancer, the lncRNA LINC00467 has been confirmed to upregulate MAPK4 expression by targeting miR-18a/b-5p, consequently enhancing the proliferation, migration, and invasive capacity of cancer cells^[27]^. Furthermore, in epithelial ovarian cancer, miR-127-3p exerts a tumor-suppressive role by directly targeting and downregulating MAPK4, which inhibits cell proliferation and migration while augmenting sensitivity to chemotherapeutic agents^[28]^. These findings elucidate that MAPK4 expression is subject to intricate post-transcriptional regulation by diverse ncRNAs and is implicated in multiple signaling pathways. Conversely, the lncRNA colorectal neoplasia differentially expressed (CRNDE) drives Th17 cell differentiation in colorectal cancer^[29]^. In tumor-associated macrophages (TAMs), knockdown of the lncRNA Xist induces the polarization of M1-type macrophages towards the M2 phenotype^[30]^. Moreover, LINC01232 reduces major histocompatibility complex class I (MHC I) expression through the LINC01232/E2F transcription factor 2 (E2F2)/neighbor of BRCA1 gene 1 (NBR1)/histocompatibility complex class I (MHCI) axis, inhibiting CD8+ T cell activity^[31]^. Overall, these studies demonstrate that ncRNAs exert broad and multilayered regulatory effects on immune cell function and tumor immune escape. Indeed, ncRNAs represent critical modulators of the cancer–immunity axis. Accordingly, targeting ncRNA-mediated networks may offer a promising strategy to overcome major barriers in immunotherapy and improve therapeutic outcomes.

In anti-tumor immune responses, CD8+ T cells directly kill tumor cells by releasing perforin and granzyme^[32]^; natural killer (NK) cells recognize and eliminate malignant cells via surface-activated receptors [e.g., natural killer group 2, member D (NKG2D)]^[33]^; and dendritic cells (DCs) activate T cells through antigen presentation, triggering adaptive immunity^[34]^. However, the TME suppresses the function of these effector cells through immune checkpoint molecules (e.g., PD-L1), inhibitory cytokines [e.g., interleukin (IL)-10, TGF-β], and metabolic reprogramming (e.g., tryptophan depletion), leading to immune evasion^[35]^. ncRNAs regulate immune cell activation, infiltration, and function, directly determining the intensity of the anti-tumor immune response^[36]^.

An instance is CD8+ T cell activation. LINC01198 enhances interferon responses (type I/II) through activation of the Nuclear factor NF-kappa-B p65 subunit (p65)/NF-κB signaling axis, promoting antigen presentation and inflammatory cytokine secretion, significantly boosting CD8+ T cell-mediated tumor cell killing^[37]^. Suppression of circular FAT1 (circFAT1) increases signal transducer and activator of transcription 1 (STAT1) binding to the C-X-C motif chemokine ligand (CXCL)9/CXCL10 promoters, promoting CD8+ T cell infiltration and enhancing their cytotoxic activity; knocking down circular protein tyrosine phosphatase non-receptor type 22 (circPTPN22) augments T cell infiltration and enhances anti-tumor functionality through the signal transducer and activator of transcription 3 (STAT3)-Sirtuin 1 (SIRT1) interaction^[38]^.

Another case is NK cell Cytotoxicity. Certain miRNAs regulate NK cell activity by targeting NKG2D and its ligands, major histocompatibility complex class I chain-related proteins A and B (MICA/B). For instance, miR-30c enhances NKG2D activity by inhibiting homeobox containing 1 (HMBOX1), while miR-93 reduces its expression^[39]^. Circular FOXO3 (circular FOXO3) sponges miR-29a-3p and miR-122-5p, relieving their inhibition of NK cell activity. Overexpression of this circRNA significantly enhances NK cell-mediated cytotoxicity against cancer cells^[40]^.

ncRNAs have the abilities to modulate the immune-suppressive microenvironment, such as reversing T cell exhaustion and enhancing therapeutic sensitivity^[41]^. Regarding regulatory T cell (Treg) infiltration, miR-214 is delivered to T cells via exosomes, inhibiting phosphatase and tensin homolog (PTEN) and promoting Treg cell expansion^[42]^. miR-21 enhances inducible T-cell COStimulator (ICOS)/ICOS ligand (ICOSL)-dependent endothelial-Tregs interactions^[43]^. miR-182 induces forkhead box P3 (FOXP3)/TGF-β/IL-17 expression^[44]^, and large intergenic non-coding RNA POU3F3 (linc-POU3F3) drives mothers against decapentaplegic homolog 2/3 (SMAD2/3) phosphorylation, promoting Treg cell expansion^[45]^.

Nonetheless, another line of research suggests that, in the liver, miR-21 may not function exclusively as a conventional oncogenic miRNA. In diethylnitrosamine-induced and PTEN-deficient mouse models of hepatocellular carcinoma (HCC), total or hepatocyte-specific ablation of miR-21 paradoxically promoted hepatocarcinogenesis, an effect associated with complex molecular alterations and changes in inflammatory/immune anti-tumoral responses, thereby challenging the prevailing view of miR-21 as a uniformly pro-tumorigenic factor in HCC^[46]^.

ncRNAs are implicated in the signaling pathways mediating resistance to cancer immunotherapy. Studies have demonstrated that low expression of miR-15b-5p impairs the therapeutic efficacy of PD-1 inhibitors via upregulating PD-L1, indicating that the loss of miR-15b-5p expression may be a critical determinant of resistance to PD-1 inhibitors^[47]^. The miR-200/zinc finger E-box binding homeobox 1 (ZEB1) module within the epithelial-mesenchymal transition (EMT) regulatory axis can directly upregulate PD-L1, thereby reducing resistance to ICIs^[48]^.

## ncRNAs IN ICIs

### Mechanisms of ICIs

Under physiological conditions, the immune system is capable of recognizing and clearing tumor cells. However, during tumor progression, tumors develop multiple mechanisms to evade detection and elimination by the immune system, ultimately resulting in advanced-stage disease^[49]^. One of the immune evasion mechanisms is immune checkpoint signaling, which mediates self-tolerance and protects normal tissues from immune cell attacks. Immune checkpoints also contribute to the immune-suppressive TME by promoting the generation of Tregs, producing immunosuppressive cytokines and chemokines. During tumor formation, immune checkpoints are activated within tumor cells, enhancing immune resistance and mediating tumor immune evasion^[50]^. Additionally, interactions between immune checkpoint molecules and their ligands suppress T cell function, complicating the physiological immune response against tumor-associated antigens (TAAs). Immune checkpoints and their ligands are frequently upregulated in the TME of various human malignancies, posing significant barriers to effective anti-tumor immune responses^[51]^.

The most well-established immune checkpoints comprise PD-1 and its ligand PD-L1, as well as cytotoxic T-lymphocyte antigen 4 (CTLA-4). Additional key checkpoints, including lymphocyte-activation gene 3 (LAG-3), T-cell immunoglobulin and mucin domain-3 (TIM-3), CD47, T-cell immunoglobulin and immune receptor tyrosine-based inhibition motif (ITIM) domain (TIGIT), and V-domain Ig suppressor of T cell activation (VISTA), have also been validated and characterized^[52]^. These molecules serve as core mediators in the regulatory network of immune activity, ensuring that the immune system mounts an effective defense against pathogens while preventing off-target damage to normal host tissues^[53]^.

#### PD-1 signaling

PD-1, also designated as CD279, is abundantly expressed on the surface of activated T lymphocytes, B lymphocytes and macrophages, and acts as a pivotal immune regulatory molecule. It exerts a core function in sustaining immune tolerance and preventing the onset of autoimmunity^[54]^. As a transmembrane receptor, PD-1 binds to two cognate ligands, PD-L1 and programmed cell death ligand 2 (PD-L2). This ligand-receptor interaction triggers antigen-specific T cell apoptosis (programmed cell death), while simultaneously reducing apoptosis in Tregs, a subset of anti-inflammatory and immunosuppressive T cells^[55]^. PD-L1 is broadly expressed on the surface of diverse hematopoietic and non-hematopoietic cells. By contrast, PD-L2 exhibits a much more restricted expression pattern, and is mainly detected on macrophages, DCs, and specific non-hematopoietic cells in tissues including the lung^[56]^. PD-1 is rapidly expressed on T cells following their activation. The cytoplasmic tail of PD-1 harbors two tyrosine-based structural motifs: the ITIM with the consensus sequence (V/L/I/XpYXX/L/V), and the immune receptor tyrosine-based switch motif (ITSM) with the consensus sequence (TXpYXXV/I)^[57]^. The binding of T cell-expressed PD-1 to tumor cell-expressed PD-L1 inhibits the phosphatidylinositol 3-kinase (PI3K)-Akt and Rat sarcoma virus protein (Ras)-rapidly accelerated fibrosarcoma protein (Raf)-mitogen-activated protein kinase kinase (MEK)-extracellular signal-regulated kinase (ERK) signaling cascades, thereby suppressing the proliferation and differentiation of effector T cells, and blocking the cytotoxic T lymphocyte-mediated anti-tumor immune response^[58-61]^.

#### CTLA-4 signaling

CTLA-4 is a type I transmembrane glycoprotein of the immunoglobulin superfamily which has been widely reported in CD4+ and CD8+ T cells. It is considered a negative regulator of anti-tumor immunity. Structurally, CTLA-4 shares significant homology with the co-stimulatory molecule CD28. It binds to CD80 (B7-1) and CD86 (B7-2) on antigen-presenting cells (APCs) with higher affinity than CD28, thereby suppressing the activity of cytotoxic T cells and ultimately promoting tumor cell immune evasion^[62]^. CTLA-4 is typically expressed after T cell activation and plays a role in downregulating or blocking T cell activation^[63]^. The cytoplasmic domain of CTLA-4, while similar to that of CD28, lacks intrinsic catalytic activity. However, the intracellular domain of CTLA-4 contains a unique tyrosine-valine-lysine-methionine (YVKM) motif, which can trigger inhibitory signaling^[51]^. During the induction phase of anti-cancer immune responses, CTLA-4 on T cells inhibits the formation of interactions between CD80/CD86 on APCs and CD28, delivering inhibitory signals that directly suppress T cell activation^[64]^. Furthermore, T cell receptor interaction molecules (TRIM) bind to CTLA-4, acting as a chaperone that facilitates its ultimate transport to the cell membrane. The CTLA-4 signaling can lead to the downregulation and dysfunction of T cells^[53,65]^.

### ncRNA-mediated regulation of immune checkpoint molecules

As research into immune checkpoint molecules continues to advance, inhibitors targeting these molecules have been successfully applied in clinical settings^[51]^. However, some patients do not experience significant benefits from ICI therapies, primarily due to the development of treatment resistance in many tumor patients^[66]^. This has driven research aimed at targeting molecules associated with ICIs to overcome such resistance. During cancer progression and development, ncRNAs dynamically regulate biological processes at both transcriptional and post-transcriptional levels, influencing tumorigenesis, invasion, metastasis and drug resistance^[67]^. Although ncRNAs do not directly encode immune-related proteins, they play a crucial role in modulating various aspects of immune responses, including antigen presentation, immune cell differentiation, and immune infiltration^[68-70]^. When regulating these processes, certain ncRNAs contribute to the formation of an immunosuppressive microenvironment, thereby promoting immune evasion by tumor cells. Therefore, understanding the emerging role of ncRNAs in regulating immune checkpoints is vital for advancing cancer immunotherapy research.

#### ncRNA-mediated regulation of PD-1/PD-L1 expression

**Figure 1:**
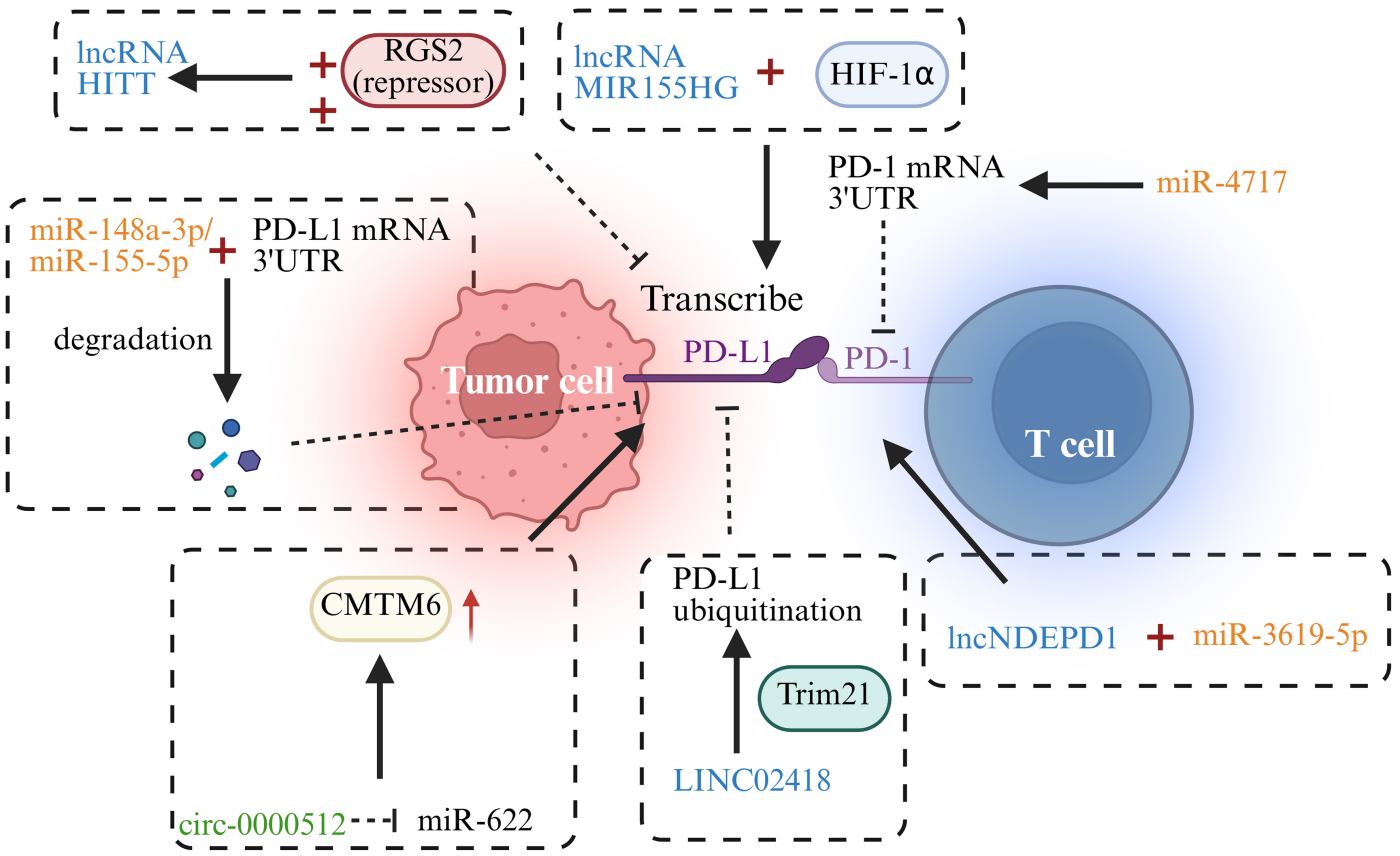
ncRNAs in PD-1/PD-L1-related pathways. →: Solid arrows indicate positive regulation, activation, or promotion of downstream biological processes. ⊣: Blunt-ended lines represent inhibitory effects or negative regulation. --→: Dashed arrows indicate indirect regulation or putative mechanisms that are supported by existing evidence but not fully elucidated. ↔: Bidirectional arrows denote reciprocal interactions or feedback regulation. Color-coded elements are used to distinguish tumor cells, immune cells, ncRNAs, and therapeutic modalities, as indicated in each panel. Created in BioRender. Zhang, D. (2026) https://BioRender.com/qpgfbzl. ncRNA: Non-coding RNA; PD-1: programmed cell death protein 1; PD-L1: programmed death-ligand 1; lncRNA: long non-coding RNA; HITT: hypoxia-inducible factor 1-alpha inhibitor at translation level; RGS2: regulator of G protein signaling 2; HIF-1α: hypoxia-inducible factor 1-alpha; mRNA: messenger RNA; 3′ UTR: 3′ untranslated region; CMTM6: CKLF-like MARVEL transmembrane domain containing 6.

The role of ncRNAs in tumor immune evasion is becoming increasingly evident, particularly through their regulation of the PD-1/PD-L1 signaling [Figure 1]. Several ncRNAs modulate PD-L1 expression by directly interacting with its transcription factors or the promoter regions of its gene, thereby affecting PD-L1 transcription levels^[71]^. Specifically, certain lncRNAs regulate PD-L1 expression by binding with transcription factors, either activating or suppressing its expression^[72]^. For instance, lncRNA MIR155 host gene (*MIR155HG*) binds to hypoxia-inducible factor 1-alpha (HIF-1α), promoting the active transcription of the PD-L1 gene, which leads to its overexpression and enhances tumor cell immune evasion in HCC^[72]^. In contrast, lncRNA HIF-1α inhibitor at translation level (HITT) recruits the inhibitory transcription factor regulator of G protein signaling 2 (RGS2) to suppress PD-L1 transcription, thereby reducing tumor cell immune evasion^[73]^. Additionally, LINC02418 can downregulate PD-L1 expression through the ubiquitination of PD-L1 mediated by the epigenetic modifier enzyme Trim21 in non-small cell lung cancer (NSCLC)^[74]^.

Many studies have demonstrated that specific miRNAs, such as miR-148a-3p, miR-155-5p, and miR-105-5p, can target the 3′ untranslated region (UTR) of PD-L1 mRNA to suppress its expression, thereby enhancing T cell immune responses and diminishing tumor cell immune escape^[75-77]^. Furthermore, interactions between different ncRNAs can also regulate PD-L1 expression. For example, the circular ncRNA circ-0000512 binds to miR-622, suppressing its activity and promoting CKLF-like MARVEL transmembrane domain containing 6 (CMTM6) expression, which subsequently reduces the ubiquitination of PD-L1, inhibiting its degradation^[78]^. lncRNA KCNQ1 overlapping transcript 1 (KCNQ1OT1) can sponge miR-15a to inhibit its activity, thereby enhancing PD-L1 expression^[79]^.

In addition to regulating PD-L1 expression, ncRNAs also influence immune escape by modulating PD-1 expression, further strengthening or weakening immune responses. Studies have shown that miR-4717 targets the 3′ UTR of PD-1 mRNA, inhibiting its expression and leading to altered immune regulation^[80]^. Moreover, the lncRNA long non-coding RNA NDEPD1 (lncNDEPD1) interacts with miR-3619-5p, inhibiting its activity and promoting PD-1 expression, which enhances tumor immune evasion^[81]^.

Indeed, the modulation of the PD-L1/PD-1 signaling by ncRNAs has become a significant area of research in understanding cancer immune escape mechanisms. Abnormal activation of the PD-1/PD-L1 pathway is a core mechanism underlying tumor immune evasion, and ncRNA-mediated regulation allows for precise modulation of this pathway. A thorough understanding of the mechanisms by which ncRNAs modulate the PD-L1/PD-1 immune checkpoint axis holds great potential for developing innovative therapeutic strategies for cancer immunotherapy. For instance, targeting specific miRNAs or lncRNAs may help suppress PD-L1 expression, restore T cell immune function, and enhance the effectiveness of ICI therapies.

#### ncRNA-mediated regulation of CTLA-4 expression

**Figure 2:**
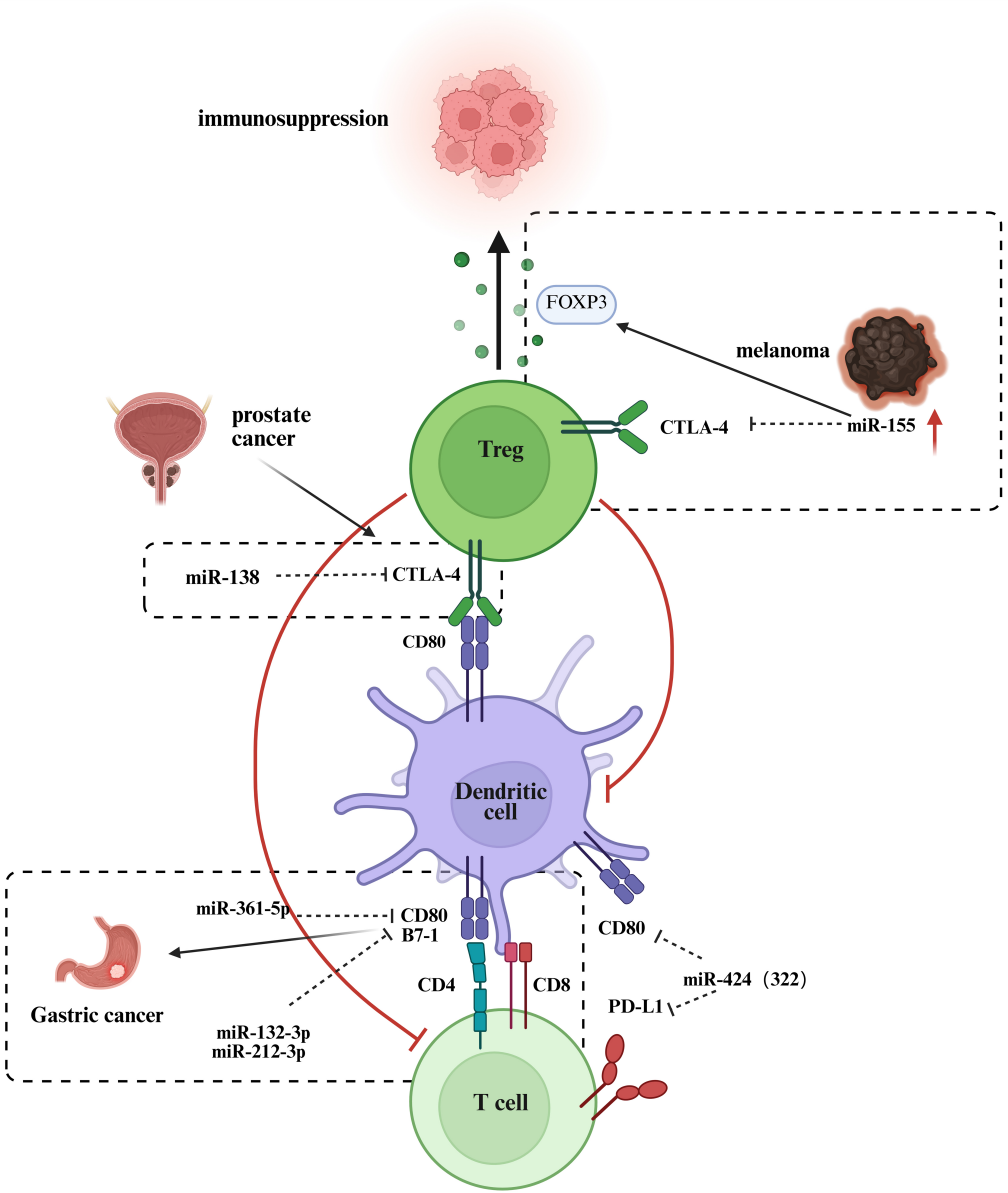
ncRNAs in CTLA-4-related pathways. →: Solid arrows indicate positive regulation, activation, or promotion of downstream biological processes. ⊣: Blunt-ended lines represent inhibitory effects or negative regulation. --→: Dashed arrows indicate indirect regulation or putative mechanisms that are supported by existing evidence but not fully elucidated. ↔: Bidirectional arrows denote reciprocal interactions or feedback regulation. Color-coded elements are used to distinguish tumor cells, immune cells, ncRNAs, and therapeutic modalities, as indicated in each panel. Created in BioRender. Zhang, D. (2026) https://BioRender.com/ain6mb0. ncRNAs: Non-coding RNAs; CTLA-4: cytotoxic T-lymphocyte–associated protein 4; FOXP3: forkhead box P3; Treg: regulatory T cell; PD-L1: programmed cell death ligand 1; TCR: T cell receptor; DC: dendritic cell.

CTLA-4 is a critical immunosuppressive molecule within the immune system. It plays a central role in regulating immune responses, maintaining immune tolerance, and preventing autoimmunity. CTLA-4 exerts its effects by binding to ligands of the B7 family, thereby inhibiting T cell activation and proliferation, which restricts excessive immune activation^[82]^. Recent studies have highlighted the significant role of miRNAs in the regulation of CTLA-4 expression and function [Figure 2]. For example, miR-138 binds to the 3′ UTR of CTLA-4 mRNA, inhibiting its expression in CD4 T cells, which enhances T cell activation and function^[83]^. This mechanism suggests that miR-138 may enhance immune responses by promoting the activity of effector T cells, playing an important role in specific immune reactions. Furthermore, in metastatic melanoma, the expression of CTLA-4 on Treg cells is significantly suppressed by miR-155-mediated post-transcriptional silencing, accompanied by an upregulation of FOXP3 expression, which collectively enhances the proliferation and immunosuppressive function of Treg cells^[84]^. The study also confirmed that this low-expression state is closely associated with poor prognosis in patients and a reduction of CTLA-4 protein in circulating Treg cells, revealing a novel mechanism by which the TME promotes immune evasion through miR-155^[84]^.

Moreover, miRNAs also influence the progression of immune responses by regulating the expression of CD80, the ligand for CTLA-4. CD80 is a crucial co-ligand that binds to its corresponding receptors, CD28 or CTLA-4^[64]^. CD80 plays a significant role in various tumor types, with low expression contributing to immune evasion, while high expression is closely associated with tumor cell migration, invasion, and immune escape mechanisms^[85]^. The expression level of CD80 correlates with the malignancy of the tumor, patient prognosis, and the effectiveness of immunotherapy. For instance, high CD80 expression in lung adenocarcinoma is indicative of a better prognosis, whereas it is linked to poorer outcomes in breast cancer and cutaneous squamous cell carcinoma^[85]^. Studies have shown that polymorphisms in the 3′ UTR of the B7-1 gene (*CD80*) [single nucleotide polymorphism (SNP) rs1599795 A > T] significantly increase the risk of gastric cancer. This polymorphism weakens the interaction between miR-361-5p and B7-1, while enhancing the binding of miR-132-3p and miR-212-3p to B7-1, leading to the downregulation of B7-1 and promoting cancer progression^[86]^. CD80 can also be expressed on tumor cells, where its interaction with CTLA-4 weakens T cell-mediated cytotoxicity against tumor cells^[87]^. A study revealed that miR-424 (322) targets the 3′ UTRs of PD-L1 and CD80, downregulating both, thereby promoting CD8 T cell proliferation and infiltration after chemotherapy and reversing chemotherapy resistance^[88]^.

These findings underscore the important role of miRNAs in regulating both the CTLA-4 and PD-1/PD-L1 pathways. Beyond modulating the expression of immune checkpoint molecules, miRNAs can affect the activation, differentiation and function of immune cells, thereby regulating the magnitude and direction of immune responses^[89]^. Future studies are expected to uncover additional miRNAs associated with CTLA-4 and the B7 family, providing a deeper understanding of their role in tumor immune evasion and offering potential targets for more effective clinical immunotherapy interventions.

### ncRNAs as biomarkers for ICI therapy

Abnormal expression of ncRNAs plays a crucial role in immune therapy responses and prognostic assessments in various cancers, making them promising biomarkers. In breast cancer, expression of the lncRNA TCL6 is closely associated with immune cell infiltration and patient survival, suggesting its potential as a significant prognostic marker^[90]^. Moreover, the level of circRNA-002178 in plasma exosomes from lung adenocarcinoma patients correlates positively with tumor malignancy. Overexpression of circRNA-002178 not only promotes cancer cell proliferation and metastasis but also facilitates early diagnosis through non-invasive detection methods, offering a novel approach for clinical practice^[23]^. The lncRNA MIR155HG has been characterized in pan-cancer studies, which revealed a robust positive correlation between its elevated expression and the expression levels of canonical immune checkpoint regulators (PD-1, PD-L1 and CTLA-4), as well as the abundance of tumor-infiltrating immune cells. This finding supports its potential utility as a pan-cancer prognostic biomarker^[91]^. This finding suggests that ncRNAs play a dual role in immune evasion: not only as regulatory factors involved in immune checkpoint modulation, but also as crucial molecular markers for predicting the efficacy of immune therapies.

Furthermore, certain miRNAs (e.g., miR-21, miR-155) and lncRNAs [e.g., HOTAIR, metastasis-associated lung adenocarcinoma transcript 1 (MALAT1)] in lung cancer regulate signaling pathways closely associated with cancer initiation and progression, influencing the development of therapy resistance. This provides support for the development of personalized treatment strategies^[92]^. Overall, ncRNAs not only influence tumor immune escape through their involvement in immune checkpoint regulation but also serve as valuable tools for predicting immune therapy efficacy and patient prognosis based on their expression profiles. This emerging molecular framework provides a valuable resource for precision medicine and opens new avenues for future cancer research and treatment strategies.

## ncRNAS IN CANCER IMMUNE THERAPY

As pivotal regulators of the tumor immune microenvironment, ncRNAs mediate key biological processes across diverse cancer immunotherapies by targeting pathways governing immune cell differentiation, functional activation, and tumor immune evasion. Specific ncRNA regulatory networks underlie both immune cell-based therapies - including T-cell, macrophage, and DC approaches - and non-cellular modalities such as cytokine therapy, oncolytic virotherapy, and cancer vaccines. These ncRNAs reverse immune cell exhaustion by targeting immune checkpoints, remodel immune cell polarization to augment antitumor activity, or modulate cytokine secretion to refine the therapeutic microenvironment. Thereby, they form a unifying regulatory backbone across distinct therapeutic strategies, offering promising targets for overcoming immunotherapy bottlenecks and enhancing clinical efficacy.

### Types and mechanisms of cancer immune therapy

In the past decade, cancer therapy has undergone a phase of drastic development. Tumor therapies based on immune cell modifications have been applied in clinical research with promising outcomes. Immune modulation therapy, an essential component of cancer immunotherapy, aims to enhance anti-tumor immune responses by regulating the immune system′s function. This therapy can be categorized into various types, including ICIs, T-cell therapy, macrophage therapy, DC therapy, and non-immune cell therapies, each with specific mechanisms of action.

#### T-cell immunotherapy

T-cell-based immunotherapy can be divided into three main types based on the source of T cells and subsequent genetic or non-genetic modifications: TIL therapy, TCR-engineered T-cell therapy, and CAR-T cell therapy^[93]^.

CAR-T cell therapy CAR is a synthetic cell surface receptor that consists of four major domains: an extracellular antigen-binding domain, a hinge region, a transmembrane domain, and an intracellular signaling domain^[94]^. When the CAR binds to its target antigen, it activates T cells to attack the target cells. The CAR gene is introduced into specific immune cells via viral or non-viral methods, followed by expansion and reinfusion into the patient. Currently, CAR-T cell therapy has been approved for treating lymphoid malignancies^[95]^. The initial CAR design primarily included the extracellular hinge and transmembrane domains, forming the simple CAR. However, the earlier generation of CAR-T cells exhibited poor T-cell activation and persistence, leading to limited efficacy^[96]^. With advancements in technology, later generations of CAR-T cells incorporate co-stimulatory domains, promoting T-cell activation and enhancing anti-tumor effects^[97]^. Currently, newer generations of CAR-T therapies further include cytokine receptor signaling domains or mechanisms to induce inflammatory cytokine expression, such as IL-12 or IL-18 and various strategies emerge to enhance cell avidity in CAR-T therapies^[98-101]^.

TIL therapy TILs are a heterogeneous population of immune cells present in tumor tissues, including CD8+ T cells, CD4+ T cells, B cells, NK cells, and γδ T cells^[102]^. After tumor tissue is surgically removed, lymphocytes are isolated and expanded, activated, and re-infused into the patient to eradicate tumor cells^[103]^. These cells act as an elite force mobilized by the body to penetrate tumor areas and engage in direct combat with tumor cells, demonstrating robust tumor recognition, resistance, and attack capabilities. TILs can also modulate immune responses, enhancing the attack ability of other immune cells against tumors^[103]^. TIL therapy has been proven effective in melanoma, with a reported overall response rate (ORR) of up to 49% in patients with advanced melanoma who failed first-line treatment^[104]^.

TCR-engineered T-cell therapy TCR-T therapy is a type of immunotherapy in which T cells are genetically engineered to improve their ability to recognize TAAs. This genetic modification enhances the affinity and immune combat strength of T cells, enabling them to efficiently recognize and attack tumor cells, thereby exerting a powerful anti-tumor immune effect^[105]^. Unlike traditional chemotherapy, TCR-T therapy has the potential to rapidly eliminate tumor cells and avoid the delayed effects commonly associated with vaccines and immune checkpoint therapies^[6]^.

#### Macrophage therapy

CAR-T cell therapy has delivered unprecedented clinical benefits in the treatment of hematological malignancies, yet its anti-tumor efficacy in solid tumor settings remains severely limited. Many solid tumors are considered “immune-cold” tumors, with suboptimal immune cell infiltration in the TME^[106]^. Even in tumors with higher immune cell infiltration, the immune-suppressive TME often prevents these immune cells from effectively eliminating cancer cells^[107]^. As a result, CAR macrophages have emerged as a promising alternative therapy^[108]^. Similar to CAR-T cells, CAR macrophages consist of an extracellular antigen-binding domain, a hinge region, a transmembrane domain, and intracellular signaling domains^[109]^. The intracellular domain in CAR macrophages utilizes the same CD3ζ, incorporating an immune receptor tyrosine-based activation motif (ITAM)^[110,111]^. Additionally, CAR macrophages can enhance their phagocytic function through additional signaling domains. For example, the fusion of the CD19 PI3K recruitment domain with the CAR Fc receptor gamma-chain (FcRγ) improves phagocytosis of target cells by two-fold^[112]^.

#### DC therapy

The host organism employs APCs to recognize, capture, and process tumor antigens, which are subsequently presented to T cells to activate both cellular and humoral immune responses, thereby initiating and orchestrating anti-tumor immunity^[113]^. Studies have shown that the abundance of DCs correlates with good prognosis in cancer patients and the clinical efficacy of ICIs^[114,115]^. Based on this finding, the development of cancer vaccines targeting DCs has become a hot research topic^[116]^. The first DC vaccine, Sipuleucel-T, has been Food and Drug Administration (FDA)-approved for treating metastatic castration-resistant prostate cancer, marking a significant milestone in this field^[117]^. Currently, multiple DC vaccines are actively undergoing clinical trials to explore their potential in cancer immunotherapy.

#### Non-immune cell therapies

Cytokine therapy Cytokine therapy plays an important role in regulating the immune system and enhancing host anti-tumor immune responses^[118]^. Cytokines such as IL-2, IL-12, and interferon-gamma (IFN-γ) activate effector T cells, NK cells, and macrophages, boosting their ability to kill tumor cells^[119]^. Moreover, cytokines can inhibit immune-suppressive cells in the TME, such as Tregs and TAMs, thereby improving immune evasion and enhancing anti-tumor immune responses^[120]^. Cytokines also help activate tumor-specific immune responses and promote the formation of immune memory, providing long-term protection against tumor recurrence^[121]^.

Oncolytic virus therapy OVs are viruses that specifically infect and kill tumor cells, showing great potential in cancer therapy^[122]^. Unlike traditional cancer therapies, OVs directly infect and lyse tumor cells, causing cell death and inducing immune responses against the tumor^[123]^. Researchers are currently working to overcome some of the inherent limitations of OVs, such as recognition of OVs as foreign pathogens and potential off-target effects^[123]^.

Cancer vaccine therapy Cancer vaccine therapy aims to activate the immune system to recognize and attack tumor cells. Tumor vaccines typically contain tumor-specific or TAAs that trigger specific immune responses^[113]^. By presenting tumor antigens to APCs such as DCs, these cells activate T cells, particularly CD8+ cytotoxic T cells, which then recognize and destroy tumor cells. Additionally, vaccine therapy can promote humoral immune responses by inducing antibody production, enhancing recognition and elimination of tumor cells^[124]^. With the formation of immune memory, vaccines can not only help in initial treatment but also offer long-term immune surveillance to prevent tumor relapse.

### Impact of ncRNA on immunocellular therapy

**Figure 3:**
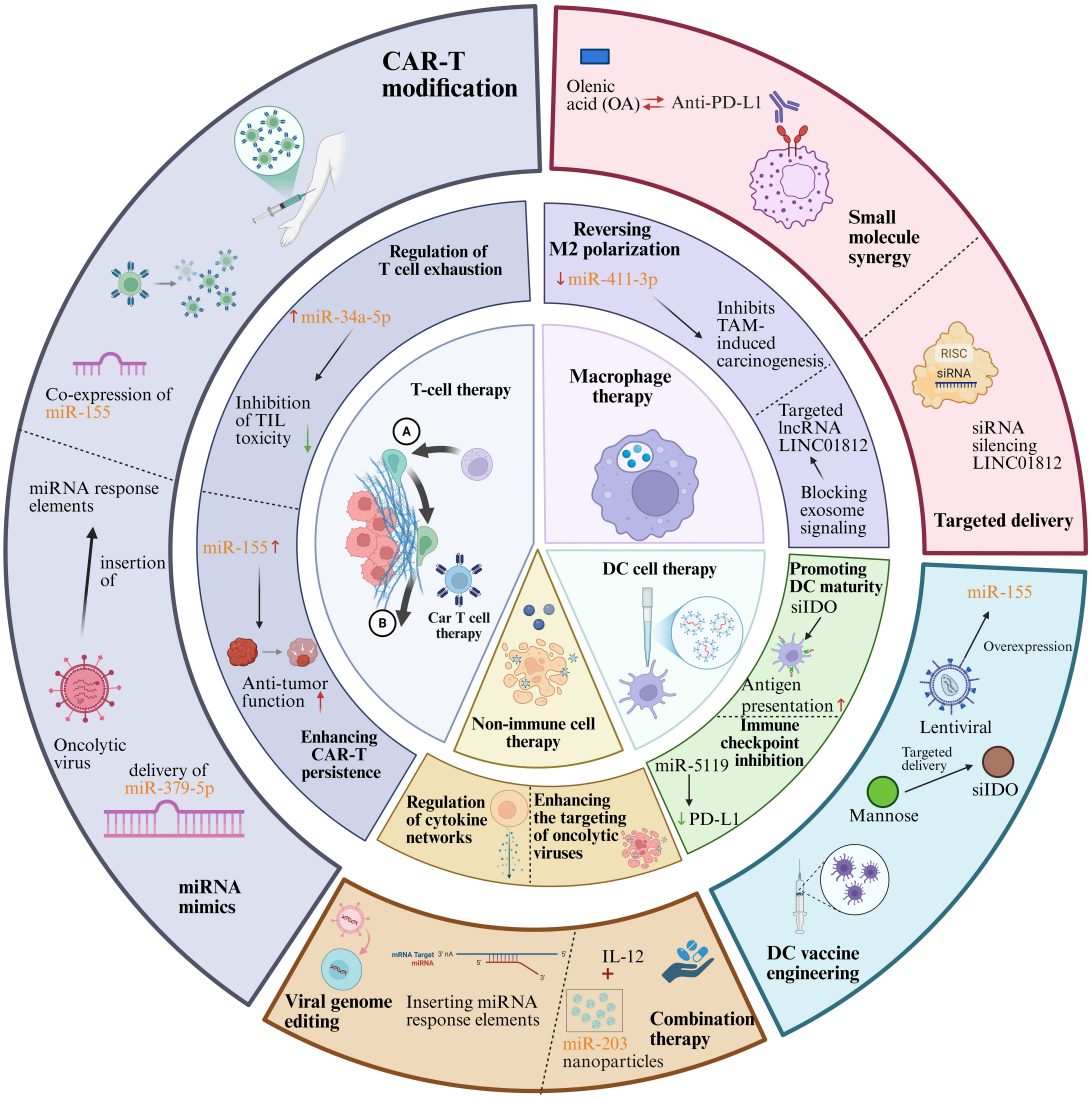
The multidimensional regulatory network of ncRNAs in cancer immunotherapies. →: Solid arrows indicate positive regulation, activation, or promotion of downstream biological processes. ⊣: Blunt-ended lines represent inhibitory effects or negative regulation. --→: Dashed arrows indicate indirect regulation or putative mechanisms that are supported by existing evidence but not fully elucidated. ↔: Bidirectional arrows denote reciprocal interactions or feedback regulation. Color-coded elements are used to distinguish tumor cells, immune cells, ncRNAs, and therapeutic modalities, as indicated in each panel. Created in BioRender. Zhang, D. (2026) https://BioRender.com/xymx1kl. ncRNAs: Non-coding RNAs; CAR-T: chimeric antigen receptor T cell; miRNA: microRNA; mRNA: messenger RNA; IL-12: interleukin-12; DC: dendritic cell; IDO: indoleamine 2,3-dioxygenase; PD-L1: programmed cell death ligand 1; RISC: RNA-induced silencing complex; siRNA: small interfering RNA; TIL: tumor-infiltrating lymphocyte; TAM: tumor-associated macrophage.

ncRNAs play a pivotal regulatory role in the differentiation of immune cells, not only by modulating gene expression to influence immune cell development but also by regulating relevant signaling pathways to control cellular function. During the differentiation of DCs, T cells, and B cells, the expression of ncRNAs is closely linked to the intensity and effectiveness of the immune response^[125-127]^. In the following sections, we further elaborate on the specific functions of distinct ncRNA subtypes (represented by lncRNAs and miRNAs) in modulating the activation and differentiation processes of immune cells [Figure 3].

#### Impact of ncRNA on T-cell therapy

In a formally registered clinical trial (NCT00287131), investigators collected and analyzed TIL specimens from 57 patients with metastatic melanoma, and performed a comparative analysis of their miRNA expression patterns^[128]^. The results revealed significantly elevated expression of miR-34a-5p and miR-22-3p in the TILs of non-responders. A predictive model built using a decision tree classification identified that TILs with low expression of miR-34a-5p exhibited stronger cytotoxicity. Furthermore, overexpression of miR-34a-5p and miR-22-3p *in vitro* suppressed the cytotoxicity of TILs^[128]^. On the other hand, miR-379-5p expression was downregulated in exhausted CD8 T cells, showing a negative correlation with TIL exhaustion in advanced tumors^[129]^. miR-379-5p targets the 3′ UTRs of the immune checkpoint proteins TIM3 and TIGIT, inhibiting their expression, thereby promoting the differentiation of CD8 T cells into memory-like effector T cells and enhancing their cytotoxicity. *In vitro* experiments demonstrated that CD8+ T cells overexpressing miR-379-5p exhibited increased antitumor activity, and in mouse models, they showed higher cytotoxicity against B16F10-Ovalbumin (B16F10-OVA) tumors. Moreover, autologous T cells from breast cancer patients transduced with miR-379-5p significantly improved their tumor-killing ability against patient-derived tumor organoids^[129]^.

The role of ncRNAs in CAR-T therapy has also garnered attention. Zhang *et al.* investigated the effects of co-expressing miR-155 or LSD1 shRNA with anti-CD19 CAR-T cells, finding that upregulation of miR-155 or downregulation of LSD1 enhanced the antitumor function of CAR-T cells^[130]^. Cytokines and the miRNA axis play a crucial role in CAR-T cell function. Yang *et al.* explored the role of IL-7 in enhancing the proliferation and antitumor efficacy of anti-CD19 CAR-T cells^[131]^. The results indicated that IL-7 significantly promoted CAR-T cell proliferation, increased the proportion of CD4+ CAR+ cells, and enhanced the S-phase of the cell cycle. Moreover, IL-7-enhanced CAR-T cells exhibited improved antitumor effects in the NAMALWA xenograft mouse model. Further studies showed that IL-7 regulates the expression of CDKN1A through miRNA-98-5p, thereby augmenting the proliferative capacity of CAR-T cells.

#### Impact of ncRNA on immune therapy via macrophage polarization

In the early stages of tumor development, macrophages accumulate in the tissues surrounding the tumor, inducing a Treg response that promotes immune evasion, EMT, and enhances the infiltration and dissemination capabilities of tumor cells^[132]^. TAMs can be categorized into two subtypes: M1-TAMs and M2-TAMs. M1-TAMs possess pro-inflammatory and anti-tumor properties, whereas M2-TAMs have anti-inflammatory and pro-tumor characteristics. Notably, M2-TAMs are associated with poor therapeutic outcomes in cancer patients^[133-135]^. Furthermore, TAMs, a core cellular component of the TME, play a critical part in facilitating tumor immune evasion, EMT, pathological tumor angiogenesis and the formation of an immunosuppressive milieu, which is mainly mediated by their functional polarization to the alternatively activated M2 phenotype^[136]^. Studies have shown that knockdown of miR-411-3p upregulates the expression of matrix metalloproteinase 7 (MMP7), promoting M2 macrophage polarization and facilitating malignant progression of colorectal cancer; conversely, overexpression of miR-411-3p reversed this process, inhibiting tumor progression and immune suppression^[137]^. This finding underscores the significant role of microenvironmental factors in macrophage polarization and their impact on tumor progression. Additionally, in a hypoxic microenvironment, HCC cells utilize exosomal miR-130b-3p to regulate the PTEN-PI3K-protein kinase B (Akt) signaling pathway, inducing M2 polarization in macrophages. Further studies revealed that oleanolic acid (OA) could inhibit this process, reduce tumor cell glycolysis, and significantly enhance the efficacy of anti-PD-1 immunotherapy, thereby broadening the therapeutic benefit of immunotherapy^[138]^. Moreover, the upregulation of certain ncRNAs has been associated with M2 macrophage infiltration in tumors. For instance, exosomes derived from cholangiocarcinoma contain the lncRNA LINC01812, which induces macrophage polarization to the M2 phenotype, thereby promoting neural invasion of cholangiocarcinoma cells^[139]^. This process significantly enhances the neuroinvasion of cholangiocarcinoma cells through the action of M2 macrophages in the TME. These findings highlight the essential role of ncRNAs in regulating macrophage polarization in tumor immune escape and progression, offering new potential targets for immunotherapy.

#### Impact of ncRNA on DC therapy

To boost the anti-tumor efficacy of *in situ* DC-based immunotherapy, Zhang *et al.* developed an innovative DC-targeting delivery system for small interfering RNA (siRNA), formally termed mannose-modified gold nanorods loaded with siRNA targeting indoleamine 2,3-dioxygenase (man-GNR-siIDO). This system uses mannose (man) as a guiding molecule to specifically target DCs and deliver siRNA against IDO. *In vivo* experiments revealed that silencing the *IDO* gene in DCs not only promoted their maturation but also upregulated the proliferation of tumor antigen-specific T cells, thereby enhancing tumor-specific cytotoxicity. In a Lewis lung cancer mouse model, combining man-GNR-siIDO with Fms-like tyrosine kinase 3 ligand (Flt3-L) therapy significantly inhibited tumor growth and delayed tumor formation^[140]^. In another study, researchers also observed a downregulation of miRNA-5119 expression in DCs from the spleens of breast cancer mice^[141]^. Microarray analysis identified that miRNA-5119 targets various negative immune regulators, including the immune checkpoint ligands PD-L1 and indoleamine 2,3-dioxygenase 2 (IDO2). Introduction of miRNA-5119 mimics into DCs effectively downregulated PD-L1 expression, alleviated T cell exhaustion, and significantly enhanced the antitumor immune response in mice. In the 4T1 breast cancer mouse model, DC vaccines engineered with miRNA-5119 mimics not only alleviated T cell exhaustion but also inhibited tumor growth^[141]^. MiR-155 plays a crucial role in regulating DC function, with its deletion impairing DC maturation, migration, cytokine production, and T cell activation. Hodge *et al.* utilized a mouse model and lentiviral transduction technology to enhance miR-155 expression in DCs to evaluate its impact on antitumor immunity. DCs overexpressing miR-155 demonstrated enhanced function in the presence of tumor antigens^[142]^. DC vaccines prepared from these miR-155 overexpressing DCs significantly improved the antitumor immune response in a breast cancer mouse model, leading to an increase in effector T cells, tumor growth inhibition, and a marked reduction in lung metastasis. In the future development of DC therapy for cancer, delivering ncRNAs, in combination with Toll-like receptor (TLR) ligands or immune-enhancing cytokines, could further unlock the therapeutic potential of DC vaccines.

### Impact of ncRNA on non-immunocellular therapy

In tumor immunotherapy, there exists a complex regulatory relationship between cytokines and ncRNAs, with both elements jointly influencing immune responses, tumor progression, and therapeutic efficacy. ncRNAs not only regulate the expression of cytokines, but cytokines can also modulate the expression of ncRNAs, thereby establishing a feedback loop that controls the tumor immune microenvironment. For instance, circular RNA NADPH oxidase 4 (circNOX4), by adsorbing miR-329-5p, upregulates fibroblast activation protein (FAP), promoting fibroblast activation and inducing the secretion of inflammatory cytokines such as IL-6. This process further contributes to the formation of a fibrotic microenvironment conducive to tumor progression in NSCLC^[143]^. Studies have shown that disruption of the circNOX4/IL-6 signaling pathway significantly inhibits tumor growth and metastasis^[144]^. Additionally, Kundu *et al.* discovered that the high expression of the miRNA-183/96/182 cluster (m96cl) in tumors modulates the transcriptional repressors forkhead box F2 (Foxf2) and zinc finger E-box binding homeobox 1 (Zeb1), altering IL-2 levels in the TME, which in turn inhibits tumor growth through a CD8+ cytotoxic T lymphocyte (CD8 CTL)-dependent mechanism^[145]^. These findings underscore the profound impact of ncRNA expression regulation on the tumor immune microenvironment.

In an IL-12 therapy experiment, IL-12 induced the expression of miR-203, which suppressed calcium/calmodulin dependent serine protein kinase (CASK), effectively inhibiting tumor growth^[146]^. In addition, studies have shown that regulatory effects of specific ncRNAs can promote the activation of immune cells, thereby boosting the therapeutic efficacy of immunotherapy. For example, miR-155, miR-142, and let-7i, in a mouse model of breast cancer, enhanced DC maturation, thereby improving the antitumor immune capability of DCs^[147]^.

In OV therapy, ncRNAs also play a crucial role. Singh *et al.* designed an OV carrying miR-148a target sites, which, when used in combination with 5-FC, significantly induced cytotoxicity in pancreatic cancer cells and delayed tumor growth^[148]^. Additionally, Jennings *et al.* employed tumor-derived extracellular vesicles (EVs) (TDEV) delivered with miR-155 or miR-19a in *in vivo* experiments to reverse the immunosuppressive effects of TAMs and enhance T-cell proliferation^[149]^.

Through the modulation of cytokines, immune cell activity, and key signaling pathways within the TME, ncRNAs significantly influence the efficacy of various therapeutic strategies, including cytokine therapy, tumor vaccines, and OV therapy. Targeted interventions aimed at ncRNAs can optimize current immunotherapy approaches, enhance antitumor immune responses, and provide novel therapeutic avenues.

## POTENTIAL APPLICATIONS OF ncRNA IN ICIS AND IMMUNOMODULATORY THERAPIES

### ncRNA as diagnostic biomarkers

**Figure 4:**
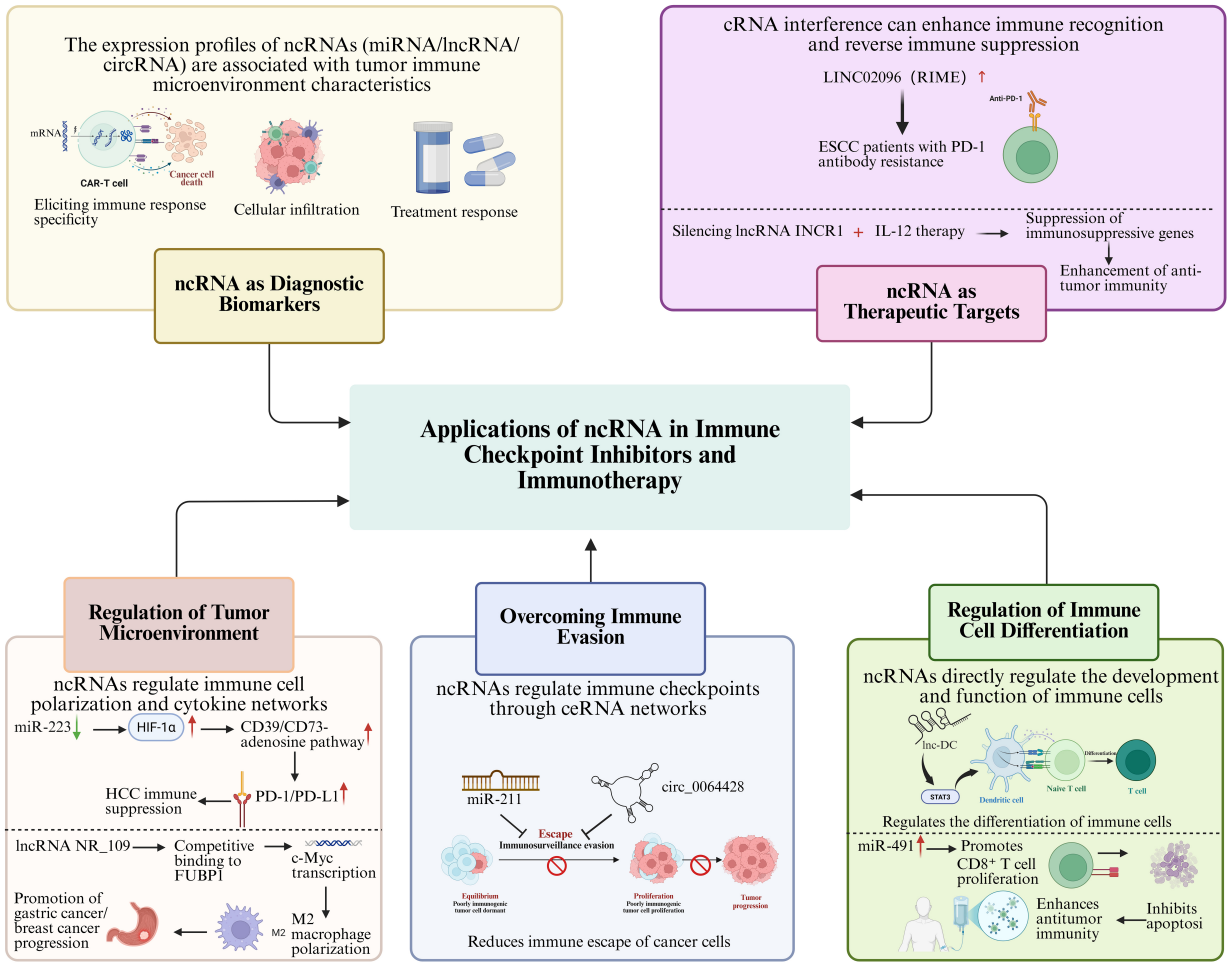
Applications of ncRNAs in ICIs and immunotherapy. →: Solid arrows indicate positive regulation, activation, or promotion of downstream biological processes. ⊣: Blunt-ended lines represent inhibitory effects or negative regulation. --→: Dashed arrows indicate indirect regulation or putative mechanisms that are supported by existing evidence but not fully elucidated. ↔: Bidirectional arrows denote reciprocal interactions or feedback regulation. Color-coded elements are used to distinguish tumor cells, immune cells, ncRNAs, and therapeutic modalities, as indicated in each panel. Created in BioRender. Zhang, D. (2026) https://BioRender.com/cwgbqbd. ncRNAs: Non-coding RNAs; ICIs: immune checkpoint inhibitors; miRNA: microRNA; lncRNA: long non-coding RNA; circRNA: circular RNA; mRNA: messenger RNA; CAR-T: chimeric antigen receptor T cell; cRNA: complementary RNA; ESCC: esophageal squamous cell carcinoma; PD-1: programmed cell death protein 1; INCR1: interferon-stimulated non-coding RNA 1; IL-12: interleukin-12; HIF-1α: hypoxia-inducible factor 1-alpha; HCC: hepatocellular carcinoma; PD-L1: programmed cell death ligand 1; FUBP: far-upstream element-binding protein; c-Myc: myelocytomatosis oncogene; ceRNA: endogenous competing RNA; STAT3: signal transducer and activator of transcription 3.

In the field of ICIs and immunotherapy, ncRNAs are increasingly recognized as important diagnostic biomarkers in both research and clinical practice [Figure 4]. ICIs work by relieving the tumor cells’ suppression of the immune system, thereby restoring antitumor immune responses. Nevertheless, this therapeutic regimen fails to deliver clinical benefits to a subset of patients, and the occurrence of irAEs further compromises the overall therapeutic outcomes^[150]^. Therefore, the development of novel biomarkers, particularly those that reflect immune responses, tumor immune evasion mechanisms, and therapeutic responses, is of critical importance. In this context, the study of ncRNAs as diagnostic biomarkers has rapidly advanced.

Mounting evidence has revealed that the expression patterns of ncRNAs are tightly correlated with the remodeling of the tumor immune microenvironment, especially key processes such as immune evasion, immune cell infiltration and tumor-related immune responses^[151-153]^. As an example, in deficient mismatch repair (dMMR) colorectal cancer, miR-148a-3p negatively regulates PD-L1 expression through direct targeting, and reduced miR-148a-3p expression is linked to higher PD-L1 abundance in tumors, highlighting its promise as a circulating biomarker for immunotherapy response prediction^[75]^. In lung adenocarcinoma, miR-155-5p negatively regulates PD-L1 expression through direct targeting, demonstrating an inverse correlation with tumor PD-L1 protein levels, thereby positioning it as a potential immunomodulatory factor and putative biomarker for immunotherapeutic strategies^[76]^. The upregulation of miR-148a inhibits the expression of DNA (cytosine-5)-methyltransferase 1 (DNMT1), leading to the upregulation of suppressor of cytokine signaling 1 (SOCS1), which suppresses the TLR signaling pathway and reduces the response of tumor-associated DCs (TADC) to TLR agonists. The use of miR-148a inhibitors (miR-148ai) to restore DNMT1 expression, combined with TLR3 activation, has been shown to improve the immune response^[154]^. A nanovaccine developed by Liu *et al.* effectively enhanced DC maturation, improved tumor immune suppression, promoted anticancer immune responses, and prolonged survival^[154]^. By detecting specific miRNAs in blood or tissues, early prediction of the efficacy of immunotherapy can be achieved, helping clinicians choose the most appropriate treatment plan. Similarly, the role of lncRNAs in tumor immune modulation is increasingly recognized. Studies have shown that lncRNAs not only regulate gene expression within tumor cells but also influence tumor immune evasion mechanisms by modulating immune cell functions^[155]^. For instance, lncRNA NF-κB interacting lncRNA (NKILA) regulates the apoptosis sensitivity of T cell subpopulations, thus altering the balance between immune-activated and immune-suppressive T cell subpopulations in the TME, facilitating immune evasion^[156]^. A prospective study enrolling 74 patients with HCC and 94 healthy controls, utilizing quantitative real-time polymerase chain reaction (qRT-PCR) along with external validation from The Cancer Genome Atlas (TCGA) and Genotype-Tissue Expression (GTEx) databases, revealed that the serum lncRNAs MALAT1 and HOXA transcript at the distal tip (HOTTIP) are significantly upregulated in HCC patients^[157]^. These lncRNAs demonstrated diagnostic utility for HCC, yielding areas under the curve (AUC) of 0.896 and 0.899, respectively, thereby surpassing the traditional biomarker alpha-fetoprotein (AFP). Furthermore, their expression levels correlated with clinical pathological features and patient prognosis. Notably, a combined detection panel incorporating MALAT1, HOTTIP, and AFP achieved an incremental elevation in diagnostic accuracy, attaining an AUC of 0.968. These findings position MALAT1 and HOTTIP as potential non-invasive biomarkers for the diagnosis and prognostic assessment of HCC, thereby furnishing novel empirical evidence to inform early screening initiatives and clinical management strategies for this malignancy^[157]^.

Additionally, circRNAs, as a novel class of ncRNAs, exhibit higher stability and resistance to degradation^[158]^, making them particularly promising as biomarkers in blood. circRNAs can regulate miRNA functions or interact with proteins to participate in tumor immune evasion and immune tolerance mechanisms^[159,160]^. By studying the expression patterns of specific circRNAs, researchers can gain in-depth insights into the tumor immune microenvironment, providing new approaches for monitoring and prognostic evaluation of immunotherapy. During treatment with ICIs, the strength of the immune response directly impacts therapeutic outcomes, and ncRNA expression patterns can reflect the immune system’s status within the TME. For instance, the upregulation or downregulation of certain circRNAs and lncRNAs in some cancer patients is closely associated with PD-1/PD-L1 expression levels, T cell infiltration density, and tumor immune evasion mechanisms^[161]^. Thus, by monitoring the expression levels of these ncRNAs, early predictions of the treatment outcomes of ICIs can be made, allowing for real-time monitoring of the patient’s immune status during therapy.

Moreover, ncRNAs can also serve as biomarkers for evaluating irAEs^[162]^. Although ICIs effectively activate the immune system to attack tumors, they may also lead to the immune system attacking normal tissues, causing various immune-related side effects. Monitoring ncRNA levels in patients’ blood or tissues can assist in the early detection of irAEs, enabling clinicians to adjust treatment strategies promptly and reduce patient risks. Evidence demonstrates that circRNAs and lncRNAs drive gastric cancer progression via multiple mechanisms, including regulating chemoresistance, immune checkpoints, angiogenesis, and metabolic reprogramming. They hold distinct translational potential as prognostic biomarkers and therapeutic targets (e.g., circRNA vaccines), with an initiated phase I clinical trial (NCT06530082) guiding their clinical translation^[163]^.

Another gastric cancer study developed an immune-related lncRNA (IRL)-based prognostic model, screening 8 core IRLs to establish a risk score and validate its overall survival predictive value (AUCs: 0.658-0.766 across multiple cohorts); nomogram integration further improved 1- to 3-year survival prediction accuracy. Notably, the low-risk group benefited more from ICIs owing to higher immune phenotype scores (IPS) and tumor mutation burden (TMB), while RNF144A-AS1, a key model molecule, was highly expressed and promoted gastric cancer proliferation and invasion via EMT activation, offering novel biomarkers for prognosis and ICI selection^[164]^.

A recent investigation systematically profiled circRNA expression in 891 patients with advanced NSCLC enrolled in two large-scale clinical trials (OAK and POPLAR), leading to the identification of an 11-circRNA signature (circRNA-Sig) and the construction of a Binary-Cox predictive model. This model demonstrated robust performance in both internal and external validation cohorts, effectively discriminating patients likely to derive benefit from atezolizumab immunotherapy. Notably, it surpassed nine previously published transcriptome-based predictive models, with patients exhibiting low circRNA-Sig scores characterized by a more active tumor immune microenvironment^[165]^.

Moreover, an eight-serum circRNA panel was developed as a non-invasive liquid biopsy biomarker for early gastric cancer detection. The panel exhibited robust diagnostic efficacy, achieving an AUC of 0.87 in the training cohort and 0.83 in the validation cohort. Multi-stage validation confirmed its tumor specificity, evidenced by postoperative downregulation and differential expression compared to other gastrointestinal malignancies^[166]^.

Based on 77 melanoma patients receiving anti-CTLA-4 monotherapy, a germline mirSNP predictive signature was constructed, capable of efficiently forecasting treatment-related grade ≥ 3 toxicity (AUC = 0.793) and tumor response (AUC = 0.842). This signature was independent of biomarkers associated with anti-PD-1/PD-L1 treatment toxicity, confirming that germline mirSNPs can serve as effective predictive biomarkers for both efficacy and toxicity in anti-CTLA-4 immunotherapy^[167]^.

Integrating multi-omic data with machine learning algorithms, a breast cancer study constructed an IRL prognostic model comprising nine lncRNAs. Validation across 17 independent cohorts revealed that patients classified as high-risk exhibited significantly shortened overall survival. The model demonstrated precision in predicting survival outcomes, response to paclitaxel chemotherapy/anastrozole-fulvestrant-gefitinib (AFG) combination therapy, and efficacy of ICIs, thereby furnishing novel biomarkers for prognostic stratification and personalized therapeutic decision-making in breast cancer^[168]^.

In summary, the application of ncRNAs as diagnostic biomarkers in ICIs and immunotherapy holds great promise. These molecules not only reflect the immune status of the TME but also provide insights into the prediction and monitoring of therapeutic efficacy and irAEs. As insights into the roles of ncRNAs in immunotherapy continue to expand, they can eventually be developed into feasible clinical diagnostic approaches, enabling more precise treatment regimens for cancer immunotherapy.

### ncRNAs as therapeutic targets

In the field of immunotherapy, ncRNAs are emerging as potential therapeutic targets, gradually becoming a frontier of research [Figure 4]. ncRNAs profoundly impact the effectiveness of immunotherapy by regulating immune cell function, tumor immune evasion mechanisms, and immune responses within the TME. Certain ncRNAs not only enhance the ability of the immune system to recognize and eliminate tumors but also suppress immune-suppressive signaling pathways, thereby optimizing immunotherapeutic strategies. As reported by Liu and colleagues, the lncRNA LINC02096 (RIME) is significantly upregulated in plasma exosomes of esophageal squamous cell carcinoma (ESCC) patients with no response to immunotherapy, and is tightly associated with unfavorable prognosis and reduced therapeutic efficacy of PD-1 antibody therapy^[169]^. Concurrently, the development of small-molecule agents targeting the intricate regulatory network governing the PD-L1/PD-1 axis has emerged as a pivotal complementary strategy to circumvent antibody resistance and intervene in this pathway^[71]^. For instance, in gastric cancer, miR-105-5p suppresses PD-L1 expression through direct targeting, and its overexpression enhances CD8+ T cell activation, thereby positioning it as a potential immunotherapeutic sensitization target amenable to indirect modulation via epigenetic agents^[77]^. In HCC, the hypoxia-induced lncRNA MIR155HG binds to interleukin enhancer-binding factor 3 (ILF3), forming a positive feedback loop with HIF-1α mRNA that upregulates PD-L1 and facilitates immune evasion; targeting this lncRNA provides a molecular rationale for reversing hypoxia-mediated immunosuppression^[72]^. lncRNA HITT synergizes with RGS2 to inhibit PD-L1 mRNA translation, thereby augmenting T-cell-mediated tumor killing; its downregulation in breast cancer correlates with elevated PD-L1 expression and unfavorable prognosis, underscoring its potential as a therapeutic target^[73]^. LINC02418 promotes PD-L1 ubiquitination and degradation, consequently enhancing the efficacy of anti-PD-L1 antibodies, suggesting that targeted intervention in this axis holds promise for improving immunotherapy responses^[74]^. Furthermore, in triple-negative breast cancer, circ-0000512 functions as a molecular sponge for miR-622, thereby relieving the suppression of CMTM6, stabilizing PD-L1, and reducing its ubiquitin-mediated degradation-a mechanism that promotes tumor immune evasion and may represent a viable therapeutic target for reversing immunosuppression by modulating protein stability^[78]^. In metastatic melanoma, elevated miR-155 expression suppresses CTLA4 by targeting its 3′ UTR, enhancing Treg-mediated immunosuppression and correlating with adverse patient outcomes; this suggests that intervening in miR-155 activity could constitute a potential strategy to improve immunotherapeutic efficacy^[84]^. A study encompassing 81 patients with American Joint Committee on Cancer (AJCC) stage III/IV melanoma employed Least Absolute Shrinkage and Selection Operator (LASSO) logistic regression to construct a predictive classifier for immunotherapy response, integrating plasma-specific miRNAs, serum lactate dehydrogenase (LDH) levels, age, and prior BRAF inhibitor (BRAFi)/MEK inhibitor (MEKi) treatment history. Through nested cross-validation, this model achieved an AUC of 0.847, effectively discriminating between responders and non-responders to immunotherapy, thereby furnishing a clinically applicable biomarker panel for liquid biopsy-based precision immunotherapy in melanoma^[170]^. In another investigation, data from 177 patients with pancreatic ductal adenocarcinoma (PDAC) in TCGA were leveraged to identify N^6^-methyladenosine (m6A)-related lncRNAs (m6A-lncRNAs), delineating two distinct molecular subtypes characterized by divergent tumor immune microenvironment profiles. A risk-scoring model incorporating 11 m6A-lncRNAs was subsequently developed via LASSO Cox regression, demonstrating independent prognostic value, reflecting immune microenvironment status, and offering guidance for immunotherapeutic stratification. Further functional experiments confirmed the anti-tumor function of long non-coding RNA TRAF3IP2 antisense RNA 1 (TRAF3IP2-AS1) in PDAC, providing promising biomarkers and therapeutic targets for prognosis assessment and personalized immunotherapy in this disease^[171]^.

Moreover, ncRNA interventions have shown potential to improve therapeutic outcomes and reduce tumor recurrence risk when combined with existing treatments such as chemotherapy, radiotherapy, or immunotherapy. For instance, Saini *et al.* found that silencing the immune-suppressive lncRNA interferon-stimulated non-coding RNA 1 (INCR1) in combination with IL-12 gene therapy, compared to PD-1/PD-L1 inhibitors alone, more effectively reduced the expression of immune-suppressive genes and significantly enhanced the anti-tumor activity of immune cells^[172]^. Additionally, Ma *et al*. developed a drug delivery system by upregulating miR-195 expression and utilizing Chlorin e6 (Ce6)-loaded nanobubbles, combined with sonodynamic therapy (SDT) and PD-1/PD-L1 immune checkpoint blockade. This approach significantly amplified anti-tumor immune responses and activated the functions of CTLs, NK cells, and DCs^[173]^.

Notable progress has been achieved in the clinical development of ncRNA-targeted therapeutics. Concurrently, ongoing advancements in delivery systems, including locked nucleic acid modifications and the application of lipid nanoparticle (LNP) technologies, are propelling ncRNA-targeted therapies toward enhanced specificity and safety profiles, thereby establishing critical technological foundations for subsequent clinical translation^[174]^.

These studies highlight that ncRNA-based interventions, as therapeutic targets, hold potential to not only regulate immune responses and suppress immune evasion mechanisms but also optimize existing immunotherapy strategies. Furthermore, when used in combination with other therapeutic modalities, they can enhance the effectiveness of immunotherapy, offering novel insights and strategies to overcome immunotherapy resistance and reduce tumor recurrence.

### Regulation of TME

In immunotherapy, the TME plays a crucial role in modulating immune cell function and influencing tumor immune evasion mechanisms. ncRNAs significantly alter the immune characteristics of the TME by regulating immune cells, cytokines, and signaling pathways [Figure 4]. Studies have shown that ncRNAs exhibit dual roles in promoting tumor immune evasion, immune suppression, and immune activation. For instance, miR-223 is highly expressed in myeloid cells, and its deficiency exacerbates the inflammation-driven onset of HCC^[175]^. Compared to wild-type mice, miR-223 knockout mice show an increase in PD-1+ T cells and PD-L1+ macrophages in tumors following diethylnitrosamine (DEN) + carbon tetrachloride (CCl_4_) treatment. Mechanistically, miR-223 regulates tumor immune suppression by inhibiting the hypoxia-inducible factor 1α-driven CD39/CD73-adenosine pathway, which in turn downregulates PD-1/PD-L1. Gene delivery of miR-223 can suppress angiogenesis and immune suppression in HCC, thereby inhibiting tumor progression^[175]^.

The lncRNA NR_109 shows elevated expression in M2type macrophages, and its silencing significantly impairs IL4induced polarization to the M2 phenotype, decreasing the capacity of these macrophages to facilitate tumor cell growth and metastasis^[176]^. Mechanistic studies have demonstrated that NR_109 competes with far-upstream element-binding protein 1 (FUBP1) for binding to aminoacyl-tRNA synthetase complex interacting multifunctional protein 1 (JVT-1/AIMP1), thereby preventing FUBP1’s ubiquitination and degradation. This leads to the activation of myelocytomatosis oncogene (c-Myc) transcription and subsequent promotion of M2 macrophage polarization. Clinical data have shown that high NR_109 expression correlates with poor clinical staging in gastric and breast cancer patients. These findings suggest that the NR_109/FUBP1/c-Myc axis plays a pivotal role in regulating TAM polarization and remodeling of the TME, thereby promoting cancer progression. In a study leveraging two independent clinical cohorts (*n* = 163), a five-circRNA prognostic signature was developed and validated, demonstrating efficacy in stratifying survival outcomes among melanoma patients receiving anti-PD-1 monotherapy^[177]^. The associated risk score exhibited significant correlations with distinct tumor immune microenvironmental profiles, thereby furnishing multicenter evidence supporting the clinical translation of circRNA-based prognostic biomarkers^[177]^. Additionally, miR-6794-5p is highly expressed in exosomes secreted by tumor cells overexpressing B cell lymphoma-w (Bcl-w), and it enhances tumor cell migration, invasion, and stemness by inhibiting the tumor suppressor SOCS1^[178]^. Studies indicate that miR-6794-5p activates the Janus kinase 1 (JAK1)/STAT3 signaling pathway, which induces polarization of human acute monocytic leukemia cell line (THP-1)-derived macrophages into M2 macrophages. The IL-10 secreted by M2 macrophages promotes tumor progression by creating an immunosuppressive microenvironment. Animal experiments further confirm that overexpression of miR-6794-5p leads to an increase in M2 macrophages and a reduction in M1 macrophages and CD8+ T cells. This study highlights the mechanism by which miR-6794-5p modulates the TME to promote malignant tumor progression. These findings provide new insights into the role of ncRNAs in the TME and offer potential therapeutic targets for future cancer immunotherapy.

### Overcoming immune evasion

In immunotherapy, ncRNAs can either inhibit or promote tumor immune evasion by regulating the expression of immune checkpoints [Figure 4]. In NSCLC, MALAT1 acts as a “sponge” that adsorbs and inhibits the anti-tumor miR-200a-3p^[179]^. When MALAT1 is inhibited, the expression of miR-200a-3p increases, leading to the suppression of its target gene PD-L1 expression. PD-L1 serves as a key immune regulatory molecule, and its expression on tumor cells can suppress T-cell activity, thus allowing tumor cells to escape from immune system attack. Therefore, MALAT1 modulates the miR-200a-3p/PD-L1 axis, influencing both tumor cell proliferation and invasion as well as enhancing immune evasion. Given the pivotal role of MALAT1 in NSCLC progression, it may serve as a potential therapeutic target for NSCLC. By inhibiting MALAT1 function or expression, the activity of miR-200a-3p can be restored, leading to downregulation of PD-L1 expression, thereby suppressing NSCLC cell proliferation, migration, and invasion while potentially enhancing the immune system’s anti-tumor activity^[179]^. However, in another study, the lncRNA MALAT1 has been characterized as exhibiting pronounced tissue- and cancer type-specific functions, a phenomenon attributed primarily to variations in its interacting protein partners, regulatory networks, and upstream signaling cascades across different malignancies^[180-183]^. In cancers such as renal carcinoma, bladder cancer, and melanoma, MALAT1 exerts oncogenic effects through multiple mechanisms: binding to the polycomb repressive complex 2 (PRC2) components enhancer of zeste 2 polycomb repressive complex 2 subunit (EZH2)/SUZ12 polycomb repressive complex 2 subunit (SUZ12) to induce histone H3 lysine 27 trimethylation (H3K27me3)-mediated silencing of tumor suppressor genes^[180,181,184]^; functioning as a ceRNA to sequester miRNAs, thereby upregulating oncogenic factors^[185-187]^; and being activated by upstream signals including FBJ murine osteosarcoma viral oncogene homolog (c-Fos) and TGF-β to initiate pro-tumorigenic pathways^[180,181]^. Conversely, in colorectal cancer and breast cancer, MALAT1 manifests tumor-suppressive properties^[182,183]^. This is mechanistically achieved through its interaction with TEA domain transcription factor (TEAD), which disrupts the YAP-TEAD pro-metastatic complex, and through synergistic regulation with PTEN. Clinical data corroborate these findings, demonstrating that low MALAT1 expression correlates with unfavorable patient prognosis in these contexts, while *in vivo* experiments have further validated its tumor-suppressive function^[182,183]^. Collectively, these findings suggest that MALAT1 functionality may be contingent upon tissue-specific molecular networks. The authors note that earlier studies reporting oncogenic roles for MALAT1 might have often been limited by small sample sizes and a lack of robust validation, whereas the more recent evidence supporting its tumor-suppressive functions is comparatively more reliable. Nevertheless, the precise role of MALAT1 in other cancer types warrants further investigation^[188]^.

Furthermore, circular RNA derived from FGFR1 (circFGFR1) is significantly overexpressed in NSCLC tumor tissues. NSCLC patients with high circFGFR1 levels display malignant characteristics including large tumor volume, lymph node metastasis and low cellular differentiation. circFGFR1 expression is correlated with poor prognosis and serves as an independent biomarker for predicting postoperative recurrence and overall survival in NSCLC patients. circFGFR1 can directly interact with miR-381-3p and serve as a molecular sponge to enhance the expression of its downstream target C-X-C chemokine receptor type 4 (CXCR4). This binding interaction does not result in the mutual degradation of the two RNAs, but forms a functional complex instead. Upregulated CXCR4 contributes to NSCLC progression and resistance to PD-1 immunotherapy. CXCR4 knockdown can offset the effects of circFGFR1 on NSCLC cell proliferation, migration and invasion. Elevated circFGFR1 expression is associated with cytotoxic T lymphocyte exclusion and anti-PD-1 therapy resistance in NSCLC, suggesting that circFGFR1 may exert immunosuppressive functions via CXCR4 upregulation, representing a promising target to overcome ICI resistance in NSCLC^[189]^.

In DLBCL, the lncRNA small nucleolar RNA host gene 14 (SNHG14) is significantly upregulated in DLBCL samples and cell lines. SNHG14 acts as a ceRNA sponge for miR-5590-3p, leading to the upregulation of ZEB1 expression. ZEB1, in turn, transcriptionally activates SNHG14 and PD-L1, forming a positive feedback loop that promotes immune evasion in DLBCL cells. Consequently, the SNHG14/miR-5590-3p/ZEB1 axis regulates the PD-1/PD-L1 pathway to facilitate immune escape. This discovery suggests that targeting SNHG14 could be a potential strategy to improve the efficacy of immunotherapy in DLBCL^[190]^. Leveraging two independent cohorts (*n* = 157), a study constructed a circRNA-based predictive signature, termed ICBcircSig, comprising circTMTC3 and circFAM117B^[191]^. This signature demonstrated robust predictive capacity for immunotherapeutic efficacy in melanoma patients across multiple validation cohorts, with AUC values ranging from 0.66 to 0.85. Mechanistically, ICBcircSig was elucidated to mediate immune evasion by sponging miR-142-5p, thereby upregulating PD-L1 expression. Notably, its predictive performance surpassed that of 20 existing transcriptomic signatures^[191]^.

### Regulation of immune cell differentiation

ncRNAs serve as key regulators in the differentiation of immune cells. By modulating gene expression and cellular signaling cascades, they control the development and functional maturation of immune cells [Figure 4].

Lnc-DC is a lncRNA that is specifically expressed in DCs. It regulates DC development, maturation, and the activation of T cell immune responses by binding to the transcription factor STAT3 and modulating its phosphorylation state^[192]^. lncRNAs also serve a pivotal function in modulating the differentiation and activation of T and B cells. Microarray analysis of CD8+ T cells has revealed that hundreds of differentially expressed lncRNAs participate in the activation, as well as the development of CD8+ memory and effector T cells. Some lncRNAs may influence CD8+ T cell function by modulating gene expression. For example, they may regulate the expression of transcription factors, cytokines, or other immune-related genes, thereby controlling T cell proliferation, differentiation, cytotoxicity, and survival. Furthermore, lncRNAs participate in the immune response of CD8+ T cells by regulating the timing, intensity and specificity of antigen-induced immune reactions. The expression of these lncRNAs is closely associated with the quality and efficacy of immune responses^[193]^.

miRNA-491 has been shown to regulate the proliferation of CD8+ T cells. Its expression is higher in activated CD8+ T cells, where it promotes T cell proliferation by targeting relevant signaling molecules. miRNA-491 also plays a role in regulating CD8+ T cell apoptosis. Timely apoptosis of CD8+ T cells during immune responses prevents excessive activation of the immune system, thereby avoiding tissue damage. Emerging evidence demonstrates that miRNA-491 prevents early apoptosis of CD8+ T cells via modulating apoptosis-associated genes including the B-cell lymphoma 2 (Bcl-2) protein family, thus sustaining the efficiency of immune responses. By regulating the proliferation and apoptosis of CD8+ T cells, miRNA-491 may influence the immune response to viruses and tumors. Overexpression or inhibition of miRNA-491 can lead to enhanced or weakened immune responses, respectively. Therefore, miRNA-491 may serve as a novel target for regulating T cell function in immunotherapy^[194]^. miR-28 significantly suppresses the expression of PD-1 in T cells, thereby relieving the inhibitory effect of PD-1 on T cell function. miR-28 also influences T cell differentiation and function by regulating cytokine secretion, such as IL-2 and IFN-γ. During T cell differentiation, overexpression of miR-28 promotes differentiation towards effector T cells, enhancing their proliferation and cytokine secretion abilities^[195]^.

## CURRENT CHALLENGES AND FUTURE DIRECTIONS

Despite the growing body of evidence supporting ncRNAs as biomarkers, therapeutic targets, and functional regulators of anti-tumor immunity, their clinical translation in cancer immunotherapy remains at an early and challenging stage. The barriers include limitations in biomarker stability and standardization, insufficient delivery efficiency and tumor specificity, off-target effects and toxicity, incomplete understanding of context-dependent functions within the tumor immune microenvironment, and the challenge of integrating ncRNA-based approaches with existing standard-of-care therapies. At the same time, rapid advances in molecular profiling, RNA engineering, synthetic biology, and precision delivery platforms are creating new opportunities to overcome these constraints. In this context, the following sections discuss some of the major current challenges and future directions of the field, with particular emphasis on ncRNA-based companion diagnostics, ncRNA-targeted therapeutics, engineered cells and vectors carrying ncRNA payloads, ncRNA-mediated remodeling of the tumor immune microenvironment, and combination strategies with established anticancer therapies.

### ncRNA-based companion diagnostics

ncRNAs have considerable potential as biomarkers for cancer diagnosis, prognosis, and treatment-response prediction, including in immunotherapy settings^[196]^. Specific expression profiles of ncRNAs in body fluids and tumor tissues can help discriminate cancer patients from healthy individuals, and their dynamic changes offer a real-time reflection of immunotherapy efficacy^[197]^. For instance, circRNAs exhibit greater stability compared to traditional biomarkers, and their expression levels are significantly correlated with response rates to ICIs^[198]^. Furthermore, miR-155 is closely linked to immune-cell functionality and may have potential as an immune-monitoring biomarker, although clinical validation remains limited^[199]^.

The clinical translation of this direction faces pivotal challenges. First, the stability and accessibility of ncRNAs in biological fluids may vary and the ncRNA content in tumor biopsy specimens can be relatively low. Plus, there are intratumoral heterogeneity, stromal admixture, and difficulty assigning ncRNA signals to specific cell types^[200,201]^. Second, existing studies are predominantly single-center with small sample sizes, and critical considerations, such as pre-analytics, sample handling, isolation methods, and assay reproducibility, remain to be rigorously addressed^[202]^. Third, there is currently a paucity of single biomarkers genuinely suitable for routine clinical disease diagnosis. Some ncRNAs exhibit similar expression patterns across multiple diseases^[203]^, which affects their reliability and applicability as specific diagnostic indicators for particular conditions.

To date, ncRNA-based biomarkers remain largely in the discovery-to-validation pipeline rather than routine companion-diagnostic use, despite the existence of some approved RNA-based diagnostic assays outside the companion diagnostic (CDx) setting^[204]^. Future efforts should focus on establishing an integrated “ncRNA fingerprint” detection system, combining features from multiple ncRNA classes to enhance diagnostic efficacy^[205]^. The development of rapid digital polymerase chain reaction (PCR) platforms could facilitate intraoperative subtyping and dynamic monitoring^[206]^. Elucidating underlying mechanisms through multi-omics integration and promoting conjunction with existing diagnostic tools will be essential for bridging the gap between research and clinical implementation.

### ncRNA-targeted therapeutics

Therapeutic agents targeting ncRNAs, including ncRNA-targeting small molecules, ncRNA mimics, and antisense oligonucleotides (ASOs), constitute an emerging frontier in cancer therapy, including potential applications in immuno-oncology. Their core principle involves specific binding to ncRNAs to modulate their expression, thereby reversing ncRNA-mediated immunosuppression or pro-tumorigenic effects^[204]^. For instance, cholesterol-conjugated miR-375 mimics exhibit enhanced stability and intratumoral delivery efficiency, targeting astrocyte elevated gene-1 (AEG-1) to inhibit HCC progression^[207]^. Members of the Let-7 family are implicated in gastric cancer progression, and Chrysin induces apoptosis and suppresses tumor growth via the H19 imprinted maternally expressed transcript (H19)/lethal-7a (let-7a)/coatomer protein complex subunit beta 2 (COPB2) axis^[208,209]^. Furthermore, the regulatory roles of other small RNAs such as snRNAs and PIWI-interacting RNAs (piRNAs) expand the repertoire of potential therapeutic targets^[210]^.

The clinical translation of this drug class is confronted with certain bottlenecks. First, delivery efficiency and targeting specificity may be inadequate^[211]^; conventional LNPs tend to accumulate in the liver, with delivery efficiency to solid tumors often falling low^[212]^. Second, off-target effects and immunogenicity are pronounced^[211]^; the multi-targeting nature of miRNAs complicates drug design^[213]^. Third, significant challenges persist in understanding RNA-protein interactions, including the difficulty of predicting interactions due to the structural diversity of RNA-protein complexes, the lack of high-throughput experimental data hampering model development, and the multifactorial regulation of these interactions^[214]^.

Future directions should prioritize optimizing delivery strategies to enhance stability and targeting specificity. Developing TME-responsive delivery vehicles could help circumvent hepatic accumulation and off-target effects^[215]^. In parallel, clustered regularly interspaced short palindromic repeats-Cas13 (CRISPR-Cas13)-based screening platforms may accelerate the identification of functionally important ncRNA targets for therapeutic development^[216]^. Ultimately, robust clinical trials will be required to define the safety, efficacy, and optimal clinical positioning of these approaches.

### ncRNAs as therapeutic payloads in engineered cells/vectors

Integrating ncRNAs as therapeutic payloads into engineered cells or delivery vectors represents a significant avenue for expanding their clinical application. Engineered cells, such as CAR-T cells and DCs, can be modified to stably express specific ncRNAs, thereby enhancing antitumor immunity^[217-219]^. Nanocarriers and EVs offer platforms for precise ncRNA delivery^[220,221]^. For example, immune-regulatory ncRNAs such as miR-155 illustrate both the therapeutic promise and the context dependence of this strategy^[222]^. CRISPR-based platforms may further enable programmable editing of ncRNA loci or regulatory circuits^[223,224]^. Additionally, poly(lactic-co-glycolic acid) (PLGA) shell-lipid core nanoparticles have been explored as vectors for pulmonary delivery of siRNA in lung cancer therapy^[225]^.

Several translational challenges remain. Delivery-system safety, manufacturing complexity, and long-term biocompatibility require further study^[226,227]^. For lipid-based systems, immune activation and safety profiles still require optimization^[212]^, and efficient intracellular trafficking and appropriate subcellular localization remain important barriers, particularly for RNA targets^[228]^. For an EV-based system, addressing source-dependent variability and setting up standardized methods for vesicle production and cargo loading will be beneficial^[229,230]^. Finally, because immune-regulatory ncRNAs often exert context- and dose-dependent effects, sustained or poorly controlled expression may impair rather than enhance antitumor responses^[231,232]^.

Future work should prioritize surface engineering and other targeting strategies to improve delivery precision, along with programmable RNA-engineering approaches that allow tighter control of ncRNA expression^[212,224]^. Combining improved homing of engineered cells with tumor-responsive delivery systems may further enhance tumor accumulation and therapeutic selectivity. Ultimately, rigorous preclinical standardization and well-designed clinical trials will be essential to establish long-term safety and efficacy.

### ncRNA-mediated regulation of the TME

The spatiotemporal heterogeneity of the TME is a core driver of resistance to immunotherapy. ncRNAs have emerged as key molecular regulators of TME remodeling by modulating immune cell function, tumor cell phenotype, and intercellular signaling pathways^[233]^. For instance, miR-155 exerts context-dependent immunoregulatory effects, enhancing DC function while sustaining Treg fitness^[199,222,232]^. ncRNAs also regulate macrophage polarization and the activation of cancer-associated fibroblasts^[234]^. Furthermore, miR-106a/20b can downregulate STAT3 levels, enhancing the antigen-presenting capacity of DCs^[235]^. Additionally, ncRNAs are involved in the regulation of immune checkpoint expression and the formation of an immunosuppressive TME^[236,237]^.

Clinical translation in this area faces certain challenges as well. To begin with, the regulatory mechanisms of ncRNAs are complex and highly heterogeneous; the same ncRNA can exert divergent functions depending on the cellular context^[238,239]^. The dynamic regulatory networks governing ncRNA expression within the TME remain poorly elucidated, and their spatiotemporal expression patterns in TILs are largely unknown^[240]^. Furthermore, the spatiotemporal heterogeneity of the TME poses significant obstacles to targeted delivery. Most existing studies rely on static snapshot analyses, and current delivery systems struggle to penetrate the immunosuppressive TME effectively^[241]^. Future research should leverage high-resolution spatial transcriptomics technologies, integrated with multi-omics approaches, to map dynamic ncRNA regulatory networks^[242]^. Clinical studies are warranted to validate the potential of modulating these networks to enhance immunotherapeutic outcomes.

### Combination strategies with standard-of-care therapies

Combining ncRNA-based approaches with standard-of-care therapies may offer a synergistic strategy to overcome resistance and enhance therapeutic efficacy^[243]^. This rationale is supported by growing evidence that ncRNAs modulate cellular pathways involved in resistance to chemotherapy, radiotherapy, targeted therapy, and immunotherapy^[244]^. Liposomal systems loaded with miR-1296 have been shown to enhance chemotherapy sensitivity in triple-negative breast cancer cell models^[245]^. In osteosarcoma, the methyltransferase-like 3 (METTL3)/long intergenic non-protein coding RNA 520 (LINC00520)/enolase 1 (ENO1) axis has been implicated in glycolysis-associated chemoresistance, suggesting that this pathway may represent a candidate target for combination treatment strategies^[246]^.

Several translational barriers remain. Optimal dosing, sequencing, and routes of administration for ncRNA-containing combinations have not been defined, and overlapping toxicities remain a concern^[204,246]^. In addition, clinical evidence for ncRNA therapeutics in cancer is still limited, with most studies remaining preclinical and only a small number of human interventional trials reported to date^[204,246]^.

Currently, few ncRNA combinations with ICIs or chemotherapy have entered clinical trials. Future research should focus on elucidating the synergistic mechanisms underlying these combinations to optimize therapeutic protocols. Developing multimodal machine learning frameworks could facilitate the construction of interpretable efficacy prediction models. Large-scale, multicenter clinical trials are essential to generate real-world evidence. Ultimately, exploring multimodal combination therapeutic systems holds promise for further improving patient outcomes.

## CONCLUSION

In recent years, the role of ncRNAs in cancer immunology has garnered increasing attention. Studies have shown that ncRNAs regulate immune checkpoint molecules, such as PD-1/PD-L1 and CTLA-4, and their signaling pathways, profoundly influencing immune evasion and therapeutic outcomes. Moreover, ncRNAs modulate T cell exhaustion, macrophage polarization, and DC function, reshaping the TME and reversing immune suppression. These mechanisms offer new perspectives on tumor immunotherapy and highlight the potential of ncRNAs as biomarkers for predicting therapeutic responses and prognosis. However, the clinical application of ncRNAs still faces certain challenges, such as low delivery efficiency, off-target effects, and immune activation. In this sense, novel delivery strategies, such as modified ncRNAs, viral vectors, LNPs, metal-organic frameworks (MOFs), and EVs, are gradually overcoming these barriers. Future research should focus on developing context-sensitive ncRNA regulation technologies, in combination with ICIs or immunotherapies, and employ modern technology, such as single-cell sequencing and machine learning, to elucidate dynamic regulatory networks, ultimately enabling the clinical translation of personalized treatment regimens. These advances not only reveal the multifaceted role of ncRNAs in tumor immunotherapy but also provide a solid foundation and practical direction for precision medicine.

## DECLARATIONS

### AI and AI-assisted tools statement

During the preparation of this manuscript, the AI tool GPT-4 (OpenAI, released 2023-03-14) and GPT-5 (OpenAI, released 2025-08-07) were used solely for language editing. The tool did not influence the study design, data collection, analysis, interpretation, or the scientific content of the work. All authors take full responsibility for the accuracy, integrity, and final content of the manuscript.

### Copyright

© The Author(s) 2026.
